# Current and Future Directions of Breast MRI

**DOI:** 10.3390/jcm10235668

**Published:** 2021-11-30

**Authors:** Margaret Houser, David Barreto, Anita Mehta, Rachel F. Brem

**Affiliations:** 1 George Washington University Hospital, Washington, DC 20037, USA; margarethousermd@gmail.com; 2 George Washington University Medical Faculty Associates, Washington, DC 20037, USA; davidbarreto80@gmail.com (D.B.); amehta@mfa.gwu.edu (A.M.)

**Keywords:** breast cancer, screening, magnetic resonance imaging, abbreviated MRI

## Abstract

Magnetic resonance imaging (MRI) is the most sensitive exam for detecting breast cancer. The American College of Radiology recommends women with 20% or greater lifetime risk of developing breast cancer be screened annually with MRI. However, other high-risk populations would also benefit. Hartmann et al. reported women with atypical hyperplasia have nearly a 30% incidence of breast cancer at 25-year follow-up. Women with dense breast tissue have up to a 4-fold increased risk of breast cancer when compared to average-risk women; their cancers are more likely to be mammographically occult. Because multiple cohorts of women are at high risk for developing breast cancer, there has been a movement to develop an abbreviated MRI (abMRI) protocol to expand the availability of MRI screening. Studies on abMRI effectiveness have been promising, with Weinstein et al. demonstrating a cancer detection rate of 27.4/1000 in women with dense breasts after a negative digital breast tomosynthesis. Breast MRI is also used to evaluate the extent of disease as part of preoperative assessment in women with newly diagnosed breast cancer, and to assess a patient’s response to neoadjuvant chemotherapy. This paper aims to explore the current uses of MRI and propose future indications and directions.

## 1. Introduction

Breast cancer is the most common cancer in women globally and the second leading cause of cancer deaths. In the United States, there are over 250,000 new cases of breast cancer annually with over 46,000 women dying of the disease [1]. Over the past two decades, the death rate from breast cancer has decreased by over 40%, a monumental achievement, due to the combination of improved detection as well as improved, increasingly targeted therapies [1]. When breast cancer is detected early, over 95% of women survive more than five years. Indeed, the detection and treatment of an earlier breast cancer is not only associated with improved outcome, but also with a lower intensity of care, meaning less aggressive therapy and less extensive surgery. Therefore, it is critical that every woman has the optimal chance at detection of early, curable breast cancer.

Initially, breast cancer screening was performed with mammography alone. However, as we increasingly understand breast cancer risk, we have been increasingly implementing risk-based screening, that is, screening based on an individual woman’s risk of developing breast cancer. The use of mammography alone is no longer be the optimal approach for the detection of earlier, more curable breast cancer. Depending on a woman’s individualized risk, optimal screening is tailored and may include mammography, ultrasound and MRI as well as exciting, emerging technologies such as ultrasound tomography [2] or abbreviated breast MRI. Even so, the mainstay of breast cancer screening remains mammography, and it is a critical and necessary component for virtually all women. 

Specific recommendations for mammographic screening vary widely. The American Society of Breast Surgeons and American College of Radiology recommend annual screening beginning at age 40 for all women at average risk of breast cancer [3,4], the American Cancer Society (ACS) says one should consider beginning screening at 40, with recommendations for annual screening at 45 to 54 and annual or biennial screening thereafter [5], and the United States Preventative Services Task Force recommends biennial screening from ages 50–74 [6]. While many varying recommendations exist, the data definitively demonstrates that annual mammography beginning at age 40 results in the most lives saved compared to other proposed screening protocols [4]. These recommendations are for women at average risk of breast cancer. In women with a first degree relative or second degree relatives with breast cancer, the ACR recommends beginning annual screening mammography 5–10 years earlier than the age that the family member developed breast cancer [4]. The ACS recommends that women at substantially increased risk, defined as a 20% lifetime risk of breast cancer or greater, undergo annual screening contrast-enhanced breast MRI in addition to annual mammography, as MRI remains the most sensitive imaging modality available for the detection of breast cancer [7]. This group of high-risk women includes those with *BRCA* 1 or 2 mutations, untested first-degree relatives of *BRCA*-positive patients, history of chest radiation for Hodgkin’s lymphoma between the ages of 10 and 30 years, and women with PalB2 mutations and other genetic syndromes associated with elevated breast cancer risk [7]. 

As the most sensitive exam, breast MRI has many clinical uses in addition to screening. This paper aims to outline and explore the current indications for breast MRI, including as a tool for screening high-risk populations, evaluating extent of disease as part of preoperative assessment in women with newly diagnosed breast cancer, and assessing a patient’s response to neoadjuvant chemotherapy. We will also propose future indications for breast MRI, such as using MRI as a biomarker for breast cancer risk and treatment response, as well as implementing abbreviated protocol breast MRI programs to expand access of screening breast MRI to those who would benefit from it.

## 2. High-Risk Screening

Mammography, the mainstay of breast cancer screening, is an excellent examination that has been critical in reducing breast cancer mortality; screening mammography has been proven to reduce mortality by 20–35% [8]. Yet, mammography is an imperfect examination. Although the overall sensitivity of mammography for the detection of breast cancer is 85% [9], the sensitivity of mammography is substantially reduced in women with dense breasts, with sensitivities as low as 45–65% [10]. In women with *BRCA* mutations, the sensitivity of mammograms is decreased and has been reported to be 33% [11], significantly lower than the overall sensitivity of mammography [9]. Because of mammography’s limitations, additional technologies have been developed to increase the detection of mammographically occult breast cancer. 

In women with dense breasts, screening breast ultrasound has been utilized to detect mammographically occult breast cancers. Importantly, studies have shown that the mammographically occult cancers detected with screening breast ultrasound are most frequently small, invasive, node-negative cancers [12,13]. These cancers are critical to detect before they advance to node-positive and possibly metastatic disease. Studies have demonstrated that screening breast ultrasound can detect approximately 25% more cancers in women with dense breast tissue than mammography alone [14]. Screening breast ultrasound, whether handheld or automated, has been increasingly incorporated in clinical practice to optimize the detection of these mammographically occult but clinically important breast cancers. There are exciting technological advances in ultrasound, such as the development of ultrasound tomography, which utilizes ultrasound and sound speed to improve both the sensitivity and specificity of breast cancer detection [2]. This, and likely other technologies, should help with the ongoing issue of high false-positive rates found with screening breast ultrasound, as well as increase the detection of mammographically occult breast cancers. 

**Figure 1:**
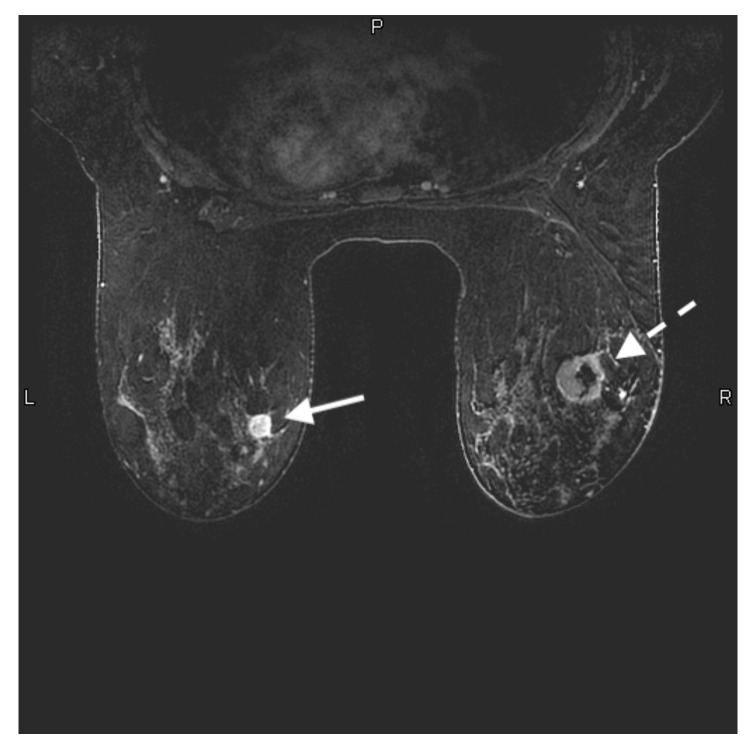
28-year-old woman with a known *BRCA* 2 genetic mutation presented for her baseline annual high-risk screening MRI. Axial contrast-enhanced T1-weighted fat-suppressed image shows a 1.4 × 1.3 × 1.2 cm rim enhancing mass in the upper inner left breast (solid white arrow). This was a biopsy-proven grade 3 invasive ductal carcinoma. There is also a 3.1 × 3.6 × 2.1 cm rim enhancing mass with central necrosis in the upper outer right breast (white dashed arrow). This was also biopsy-proven grade 3 invasive ductal carcinoma.

Women with a *BRCA* mutation have a 60–85% cumulative lifetime risk of developing breast cancer [15]. The sensitivity of MRI for the detection of breast cancer in this high-risk population ranges from 75–100% [7]. Studies have demonstrated that in *BRCA*-positive women, 44.7% of cancers are not detectable with mammography and are detected with MRI, whereas 2.1% of cancers are detected by mammogram and not identified with MRI [16]. Furthermore, MRI detected cancers in *BRCA*-positive women have a better prognosis than those detected with mammography, in that they are more frequently node -negative [15]. A study by Kriege et al. demonstrated that MRI not only detected cancers not visible with mammography and clinical breast exam, but over half were smaller than 10 mm and only 1 case of the 20 invasive cancers in this study was associated with node positivity [15]. Lo et al. demonstrated statistically significant greater cancer detection rates (CDR) with MRI when compared to mammography in high-risk women; MRI detected 21.8 cancers per 1000 women screened whereas mammography detected 7 per 1000 women screened [17]. Sippo et al. confirmed a high MRI CDR in patients with *BRCA* mutations, at 26 per 1000 women screened [18]. The robust data obtained with numerous studies investigating the use of screening breast MRI in women at markedly increased risk of breast cancer demonstrates that annual screening with MRI detects earlier, more frequently node-negative and more likely curable breast cancer. As a result, the ACS and numerous other societies agree that annual screening with MRI for women at the highest risk of breast cancer is not only recommended but strongly advised (Figure 1).

It is important to note that the population of women at increased risk of breast cancer, for whom MRI is critically important, has been expanding. Initially, the recommendation for annual screening with MRI was only for those with a genetic mutation, where 27 cancers per 1000 women screened with MRI were detected [15]. Further studies in women with a personal history of breast cancer demonstrated a CDR ranging from 10 to 29 cancers per 1000 women screened [19,20,21,22,23,24]. In addition, women with high-risk breast lesions, such as atypical lobular or ductal hyperplasia, have a CDR of 13 per 1000 women when screened with MRI [25]. Up to 30% of patients diagnosed with atypical hyperplasia develop breast cancer after 25 years [26]. Studies have demonstrated that these women also benefit with MRI screening, with a CDR of 15 cancers per 1000 women screened [18]. Sippo and colleagues reported no difference in CDR for screening breast MRI examinations performed in patients with a personal history of breast cancer (12 per 1000 examinations) or high-risk lesion (15 per 1000 examinations), compared with those performed for *BRCA* mutation or history of chest radiation (26 per 1000 examinations; *p* = 0.14 and *p* = 0.18, respectively) [18].

Women with dense breast tissue are at moderately increased risk of breast cancer (15–20% lifetime risk). Forty percent of women in the US have dense breasts [27]. Women with dense breast tissue have up to 4.6-fold increased risk of developing breast cancer when compared to average risk women [28]. In addition, the sensitivity of mammography in women with dense breast tissue is significantly decreased, with reports as low as 45%. As a result, cancers are usually larger and more frequently node-positive at the time of diagnosis [29]. Additionally, the frequency of interval cancer detection, that is, cancer detected within a year of a normal screening mammogram, is significantly higher in women with dense breast tissue. It is well-known that interval cancers have a poorer prognosis. Therefore, supplemental screening MRIs in women with dense breasts and normal mammograms has been suggested and investigated. A recent study from the Netherlands investigating the impact of interval cancers in women with dense breast tissue who underwent screening MRI and demonstrated a significant reduction in interval cancers with screening MRI [30]. Indeed, half the number of interval breast cancers were detected in women with dense breasts who underwent screening MRI; 2.5 cancer per 1000 screened with MRI, versus 5.0 per 1000 screened with mammography alone [30]. This reduction in interval cancers, which is statistically significant, demonstrates that supplemental screening MRI in women with dense breasts is beneficial. Additionally, this study demonstrated an unexpectedly high CDR when MRI was used to screen women with dense breast tissue (16.5 per 1000 screenings) demonstrating the benefit of screening MRI in women with dense breast tissue [30].

MRI screening has political implications as well. In the United States, screening mammography is covered without a co-pay or deductible as part of the Affordable Care Act (ACA); screening MRI is not. It is not uncommon for insurance to reject MRI screening, especially in women who do not have a deleterious genetic mutation. Efforts are underway to facilitate the approval of MRI screening. With the robust evidence demonstrating the significant benefit of screening MRI in women with a personal history of breast cancer, a history of high-risk lesions, and even in women with dense breast tissue, Pennsylvania Governor Tom Wolf signed Senate Bill 595 in June 2020 which requires insurance coverage of supplemental breast cancer screening with MRI in women with extremely dense breasts (BIRADS D) and in women with heterogeneously dense breast tissue (BIRADS C) and one other high-risk factor [31]. It is hopeful that additional states will follow suit and facilitate the approval of screening MRI by eliminating the financial barrier to obtaining screening MRI in women at increased risk for breast cancer.

## 3. MRI Evaluation of Newly Diagnosed Breast Cancer

**Figure 2:**
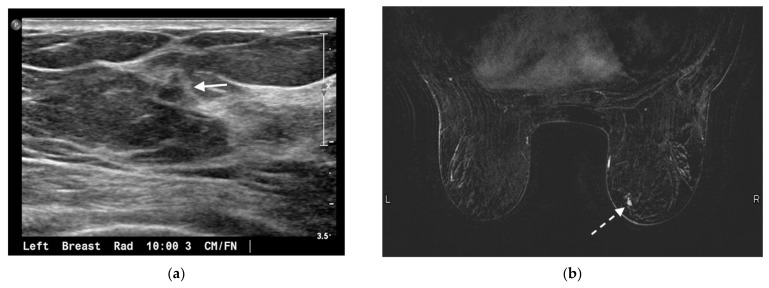
75-year-old woman presented for annual screening evaluation. Mammogram demonstrated a left breast asymmetry (not shown) which prompted further workup. (**a**) Left breast ultrasound in radial projection at the 10 o’clock axis 3 cm from the nipple demonstrates a suspicious 0.5 × 0.6 × 0.4 cm hypoechoic antiparallel mass with irregular margins and posterior acoustic shadowing (arrow), corresponding to the mammographic abnormality. Ultrasound guided biopsy yielded invasive ductal carcinoma, mucinous type. (**b**) MRI was performed to evaluate the extent of disease; axial contrast-enhanced T1-weighted fat-suppressed image demonstrates a separate 0.7 × 0.5 cm enhancing irregular mass in the lower inner quadrant of the contralateral right breast (dashed arrow). This was biopsied under MRI guidance and yielded invasive ductal carcinoma, mucinous type. The known left breast cancer is not shown.

In women with newly diagnosed breast cancer, preoperative staging is required to assess disease extent to optimize treatment strategy. Assessment of locoregional metastases into the axillary, internal mammary and supraclavicular nodal basins is critical information for surgical and treatment planning. Preoperative breast MRI plays an important role in delineating the extent of the primary tumor and diagnosing multicentric and contralateral disease, as well as in the evaluation of local nodal metastases and chest wall involvement. Studies have demonstrated that when women with newly diagnosed breast cancer are evaluated with MRI, additional foci of disease are identified in 10% of women: 7% in the ipsilateral breast and 3% in the contralateral breast [32]. Of the contralateral cancer diagnosed on preoperative MRI, most are less than 1 cm in size, and approximately one-third are ductal carcinoma in situ (DCIS) (Figure 2). 

A meta-analysis by Plana et al. [33] demonstrated that additional disease was found in the ipsilateral breast in 20% with a positive predictive value (PPV) (of lesions undergoing biopsy) of 67%, and in approximately 20%, these additional findings impacted surgical planning. Brennan et al. performed a meta-analysis of 22 studies which reported suspicious MRI detected findings in the contralateral breast of women with newly diagnosed breast cancer, and found additional foci in 9.3% of women, with a PPV (of lesions undergoing biopsy) of 47.9% yielding a cancer detection rate of 4.1% in the contralateral breast (32). In a retrospective study, Benveniste et al. demonstrated that in 76 patients diagnosed with pure DCIS who underwent preoperative MRI staging, approximately 8% had additional foci of cancer (6.6% DCIS and 1.3% invasive breast cancer), all in the contralateral breast [34].

Given its sensitivity, MRI is the most powerful tool to assess breast cancer and a significant number of women are found to have disease that was occult on mammography and ultrasound. This information aids in surgical planning with the goal of decreasing positive margins and re-excision rates. This is particularly true in women with pure DCIS or known invasive cancer with an extensive DCIS component [35]. In a cohort of 593 women who underwent breast surgery, Kuhl et al. reported that more than half of DCIS associated with invasive cancer was detected only with MRI, compared to FFDM and radiologist-performed US [35]. In the same study, positive margins were demonstrated in only 3.7% of the cases after MRI imaging and MRI-guided biopsy/bracketing, which is significantly lower than positive margin rates reported after conventional assessment in similar cohorts, ranging from 20.0–40.0% [35,36,37]. Additionally, women with invasive lobular carcinoma benefit from preoperative MRI. Lobbes et al. reported that preoperative MRI in women with ILC results in a significantly lower risk of positive margins without a concomitant increase in mastectomies [38]. 

The use of preoperative MRI in all newly diagnosed patients is still controversial and guidelines differ among institutions. Although there is solid evidence that MRI demonstrates disease that is not seen by conventional modalities, it is still unclear if that translates to better patient outcomes, specifically disease-free survival and overall survival. Moreover, studies have shown that women with newly diagnosed breast cancer who undergo MRI have a higher rate of mastectomies. Preoperative MRI may be most beneficial in patients with triple-negative breast cancer (TNBC), invasive lobular cancers, or patients who undergo breast conservation without radiation therapy. Wang et al. demonstrated that preoperative MRI improved breast cancer survival in women who underwent breast-conserving without radiation therapy [39]. Given that use of preoperative MRI is controversial, a multicenter international study with more than 7000 enrolled patients is currently underway to investigate and compare the clinical outcomes in women undergoing versus not undergoing preoperative breast MRI [40]. 

**Figure 3:**
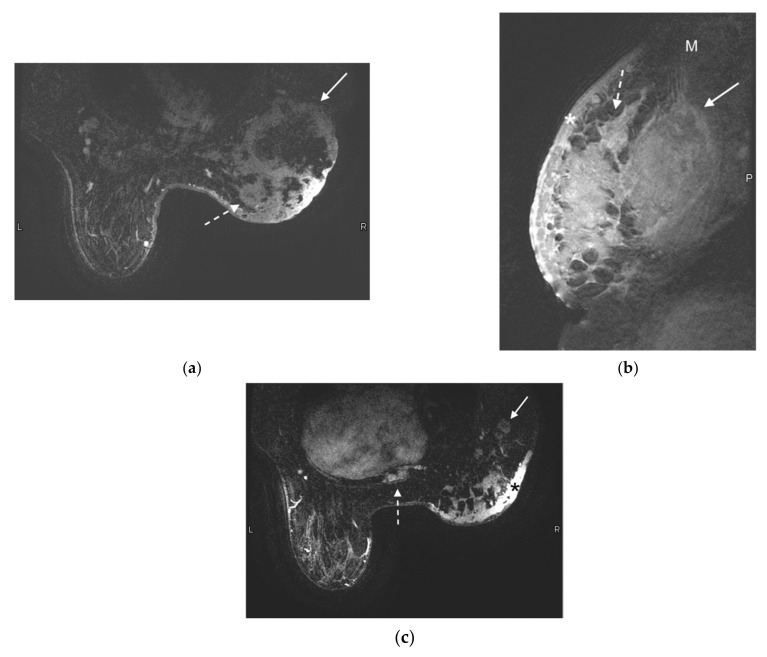
55-year-old woman presented with a palpable breast mass and asymmetric breast size. Mammogram and ultrasound showing a large mass are not shown. MRI was performed to evaluate for extent of disease. (**a**) Axial contrast-enhanced T1-weighted fat-suppressed image demonstrates a 13 cm ring-enhancing centrally necrotic lateral right breast mass (solid arrow). Additional rim-enhancing centrally necrotic satellite lesions are noted at the medial aspect of the breast (dashed arrow). (**b**) Sagittal contrast-enhanced T1-weighted fat-saturated image demonstrates a large irregular heterogeneously enhancing right breast mass invading the right pectoralis muscle (arrow). The edge of the normal pectoralis muscle (M) is seen superiorly. Diffuse skin thickening and enhancement (*) is compatible with skin involvement, and satellite lesions (dashed arrow) are redemonstrated. *p* demarcates the posterior aspect of the image. (**c**) Axial contrast-enhanced T1-weighted fat-saturated image demonstrates abnormally enlarged and enhancing right axillary lymph nodes (arrow) and an enhancing metastatic bone lesion in the sternum (dashed arrow). Right breast skin thickening (*) is again noted.

Although many trials have demonstrated improved accuracy as well as margin assessment with MRI, studies have also shown that preoperative MRI results in an increased rate of mastectomies. A meta-analysis of 19 studies by Houssami et al., reported that preoperative MRI was associated with a 1.4 increased odds ratio for mastectomy, and MRI was also associated with increased odds ratio (1.91) of a contralateral prophylactic mastectomy [41]. The increased rate of mastectomy following preoperative MRI must be considered when clinical decisions regarding preoperative MRI are made. Even so, at our institution, nearly all women with newly diagnosed breast cancer undergo MRI for assessment of extent of disease, surgical planning and reducing the need for re-excision, as we believe the benefit outweighs the risks. Figure 3 shows a preoperative MRI demonstrating extent of the newly diagnosed breast cancer. Skin and posterior chest wall involvement, as well as satellite lesions and metastatic sternal lesions, were first identified in this study, resulting in change in management. The patient received preoperative chemotherapy to attempt to reduce disease burden before surgery (Figure 3). Furthermore, at our institution the use of margin assessment tools, such as MarginProbe [42], further allows the surgical team to implement available strategies to decrease the need for re-excision.

## 4. Response to Neoadjuvant Chemotherapy

Neoadjuvant chemotherapy (NACT) is the preferable approach in patients with locally advanced (stage II or III) and TNBC. Long-term outcomes for patients receiving NACT versus adjuvant chemotherapy were demonstrated in a patient-level meta-analysis conducted by the Early Breast Cancer Trialists’ Collaborative Group [43]. When compared with adjuvant chemotherapy, NACT was associated with an increased frequency of breast-conserving surgery (65% vs. 49%) and an equivalent risk of both distant recurrence (15-year risk 38.2% vs. 38.0%) and breast cancer mortality (34.4% vs. 33.7%) [43]. However, NACT was also associated with more frequent local recurrence than adjuvant chemotherapy (15-year local recurrence 21.4% vs. 15.9%) [43]. This difference may be due to more frequent breast-conserving therapy in women with TNBC. Of note, patients undergoing NACT who achieve pathologic complete response (pCR), defined as the absence of residual invasive cancer on pathologic evaluation following surgery, have excellent long-term survival outcomes [44,45,46]. More than half of the patients with HER2-amplified tumors achieved pCR [47]. With the increasing use of NACT, methods to assess the effectiveness of neoadjuvant chemotherapy are needed. 

**Figure 4:**
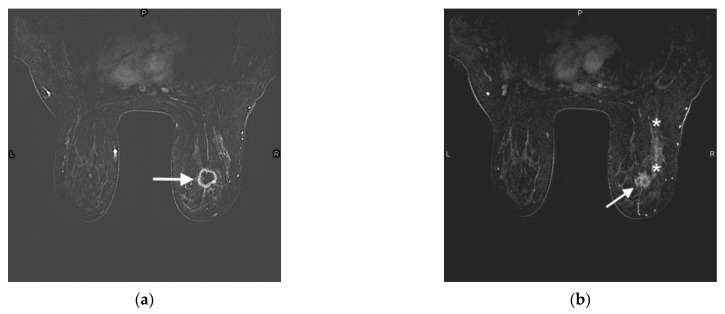

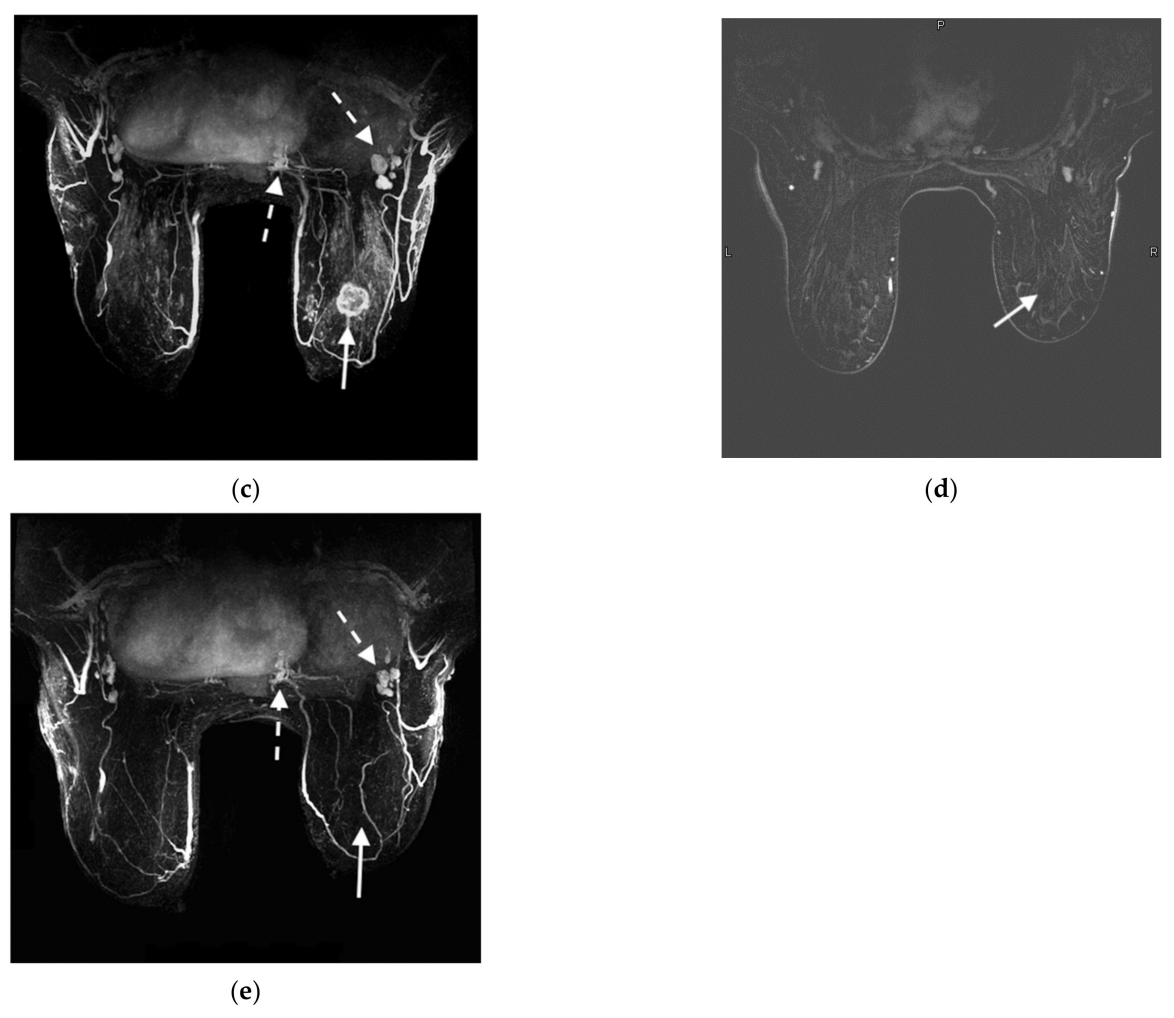
47-year-old woman with a right breast invasive ductal carcinoma. (**a**) Prechemotherapy axial contrast-enhanced T1-weighted fat-suppressed MRI shows a 2.7 × 2.5 × 2.2 cm irregular mass with a thick rim of enhancement in the upper outer right breast (arrow), compatible with the known invasive ductal carcinoma. (**b**) Prechemotherapy axial contrast-enhanced T1-weighted fat-suppressed MRI shows an area of non-mass-like enhancement compatible with DCIS (edges demarcated by (*) located posterior to the mass (arrow). (**c**) Prechemotherapy maximum intensity projection image demonstrates a right breast mass (arrow) with associated enhancing right axillary and intramammary lymphadenopathy (dashed arrows), compatible with metastatic disease. (**d**) Postchemotherapy contrast-enhanced T1-weighted fat-suppressed MRI shows resolution of the right breast mass. Susceptibility artifact from the biopsy clip is seen in the tumor bed (arrow). The area of non-mass-like enhancement has also resolved (not shown). (**e**) Postchemotherapy maximum intensity projection image demonstrates resolution of the previously seen large right breast mass (solid arrow in tumor bed). The right axillary and intramammary lymph nodes are redemonstrated but have decreased in size (dashed arrow).

It is important to assess response to NACT as early as possible during therapy, as a poor response can trigger a change in the chemotherapeutic regimen. There are multiple methods for evaluating response to neoadjuvant chemotherapy. Initially, response to neoadjuvant chemotherapy relied on physical examination along with conventional breast imaging, i.e., mammogram, and US. Studies have demonstrated a moderate correlation between pCR and physical examination, mammography and ultrasound reported at 57%, 74% and 79%, respectively [48]. More recently, studies have demonstrated greater accuracy of MRI in assessing response to NACT with a reported sensitivity of 88–89% (Figure 4) [49].

The ability of MRI to assess response to NACT is based on a number of factors, including tumor molecular subtype (less accurate in luminal, and greater accuracy in triple-negative tumors and HER-2 positive tumors) [50], as well as type of chemotherapy used. MRI has a greater likelihood of underestimating residual disease in patients treated with antiandrogenic drugs [51] and more accurate with regimens that do not include taxanes. Schrading et al. demonstrated that the use of taxanes resulted in 66.7% false-negative MRI findings in patients with residual disease, versus 20% false-negatives rate in patients treated with multiagent chemotherapy without taxanes [52]. Additionally, Chen et al. have shown that MRI can overestimate residual disease in 6–19% of cases, and underestimate in 7–28% of cases [53]. According to Reig et al., causes of overestimation of residual disease include fibrosis/treatment changes, necrotic tumor and residual benign masses, whereas tumors with nonmass morphology, tumors with nonconcentric shrinkage, antiandrogenic therapy and late-enhancing foci tend to demonstrate underestimation of residual disease [54]. MRI following neoadjuvant therapy can also be helpful in downstaging biopsy-proven metastatic axillary lymph node, with some studies demonstrating a sensitivity of 61–72% [55]. Although imperfect, MRI is currently the most accurate imaging modality to assess response to neoadjuvant chemotherapy, and particularly when response is concentric, can be quite accurate.

Recently, a novel approach to assess response to NACT has been reported. Duric and colleagues reported accurate assessment of early response to neoadjuvant chemotherapy utilizing ultrasound tomography [56]. This approach detected response early in the course of neoadjuvant chemotherapy, which may allow for earlier intervention for chemotherapeutic regimens with less-than-optimal response. Additionally, this approach does not require injection of contrast. Although additional studies are needed for further evaluation, this approach is exciting and has potential to accurately evaluate response to neoadjuvant chemotherapy early in the course of treatment.

## 5. Imaging Biomarkers

Breast cancer can be identified on MRI by its pattern of enhancement after the administration of intravenous contrast. Because malignancies undergo uncontrolled angiogenesis in patterns different from benign breast tissue, breast cancer generally appears as a relatively bright focus of enhancement on MRI. Some benign as well as high-risk lesions also enhance, and kinetic information of MRI contrast uptake and excretion from tissues can help differentiate benign and malignant lesions. It is important to note that normal fibroglandular breast tissue enhances, a phenomenon known as background parenchymal enhancement (BPE), albeit generally at a slower rate than cancer or high-risk lesions. BPE is thought to reflect the background physiologic activity of a woman’s breast tissue and is affected by each woman’s unique vascular and hormonal factors, exposure to endogenous and exogenous hormones, and use of endocrine therapy [57]. Menopausal status, menstrual cycle, hormone replacement therapy, and tamoxifen use may impact the extent of BPE in women. 

Background parenchymal enhancement is characterized in the breast imaging (BIRADS) lexicon as minimal, mild, moderate, and marked [58]. Moderate or marked BPE can hinder breast cancer detection due to the increased background uptake. Similar to mammography in a patient with dense breasts, the interpreting radiologist is looking for a bright cancer in a background of enhancement, i.e, a bright background [59]. To mitigate the decrease in sensitivity of MRI, especially in women with substantial BPE, it is recommended that women undergo MRI examinations when performed for screening during the second week (follicular phase) of their menstrual cycle, to decrease endogenous hormonal influence on their BPE [60]. 

Increased estrogen exposure is a known risk for breast cancer due to tumorigenesis via estrogen receptors. BPE is positively affected by both endogenous and exogenous estrogen. Studies have demonstrated a positive correlation between BPE and breast cancer risk [59,60]. Moderate or marked BPE is associated with a higher risk of developing breast cancer compared to women with minimal or mild BPE (odds ratio of 2.1) [61]. A study by Arasu et al. found BPE to be associated with increased risk of breast cancer independent of mammographic breast density [62]. Given these findings, it is likely that future risk models will consider including BPE in risk assessments and qualification for high-risk screening with MRI. 

Tamoxifen and other estrogen receptor modulators are used both as adjuvant therapy for estrogen receptor / progesterone receptor (ER/PR) positive breast cancer and as a risk reduction strategy in women at increased risk of breast cancer. Studies have demonstrated up to a 50% decrease in incidence of cancer when tamoxifen is used for risk reduction [63]. When MRI is performed in women undergoing tamoxifen therapy, a variable decrease in BPE is noted. Studies have begun to investigate whether the resultant decrease in BPE following tamoxifen therapy may reflect the individualized effectiveness of the therapy. It is hypothesized that the imaging manifestation of the antiestrogenic effect of tamoxifen may well be reflected in the impact on BPE. However, up to 32% [64] of patients on tamoxifen therapy do not have the expected decrease in BPE. It is possible that the lack of decrease in BPE may reflect a lesser antiestrogenic effect. Patients with little or no impact on BPE when on tamoxifen therapy may not be benefiting. Given that tamoxifen has significant side effects including deep venous thrombosis, pulmonary emboli, stroke, and endometrial cancer, the benefits may not outweigh the risks in this subset of patients. However, this is currently hypothetical and additional studies are underway to further investigate the possibility of modulating risk reduction tamoxifen therapy based on effect of BPE.

Molecular breast imaging (MBI), also known as breast-specific gamma-imaging (BSGI), is another sensitive physiologic imaging modality used to detect breast cancer [65]. This exam uses intravenous injection of ^99m^Tc-sestamibi, a nuclear radiotracer, to physiologically differentiate between benign and malignant breast tissue. MBI/BSGI detects cancer with a sensitivity and specificity of up to 96% and 80%, respectively [66] and can increase detection of mammographically occult cancers by up to 1.7% of women at increased risk of breast cancer [65]. Patients who cannot undergo MRI, whether due to obesity, inability to lay flat, MRI contrast allergy, renal insufficiency, claustrophobia, or metallic hardware incompatibility, can benefit from physiologic imaging with BSGI. 

Background parenchymal uptake (BPU) is the MBI/BSGI equivalent of BPE in MRI, and is defined as the ^99m^Tc-sestamibi uptake within normal fibroglandular tissue. Studies have shown elevated BPU is associated with increased breast cancer risk [67]; BPU can therefore act as a nuclear medicine biomarker for predicting risk. Additionally, just as tamoxifen can decrease BPE on MRI, it can also decrease BPU on MBI/BSGI. Noting a patient’s change in BPU with tamoxifen therapy may reflect the effectiveness of tamoxifen and influence treatment decisions with estrogen receptor modulators. Additional studies are needed to further define the use of assessing BPE on management decisions including tamoxifen therapy.

## 6. Abbreviated Breast MRI

Given that MRI is the most sensitive tool to detect cancer, there has been a movement to expand access to MRI screening. Due to its cost, full-protocol, dynamic contrast-enhanced breast MRI (fpMRI) has traditionally been offered as a screening tool only to women at the highest risk for breast cancer [68]. An fpMRI, which includes multiple phases of axial T1-weighted pre- and post-contrast images with and without fat suppression, axial T2 or STIR, and a sagittal delayed post-contrast sequence, takes 45–60 min to acquire, in addition to the time required to prepare and position the patient for the examination. 

**Figure 5:**
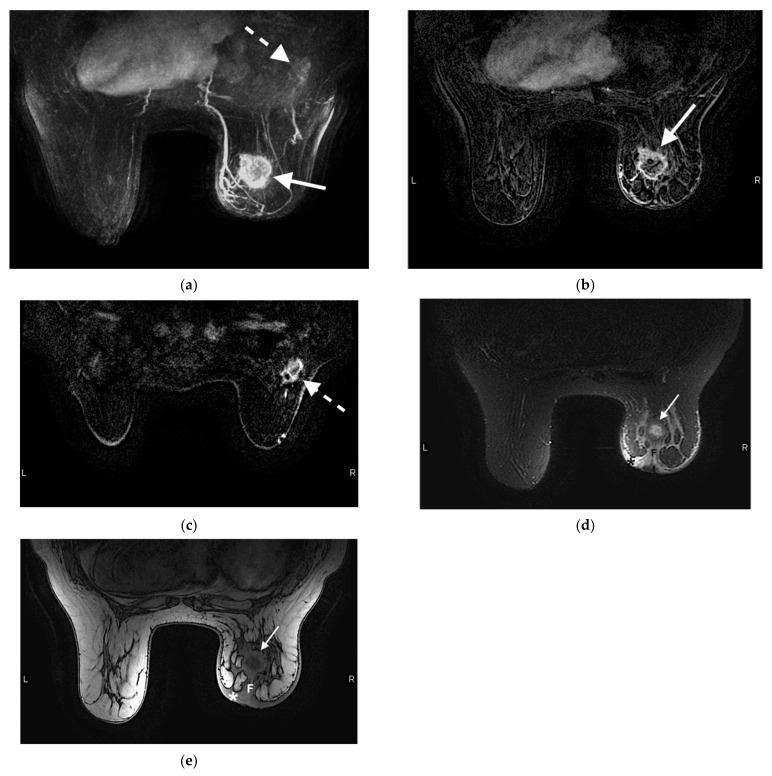
Example protocol of an abMRI. 61-year-old woman presented for screening MRI. (**a**) Maximum intensity projection image illustrates a 3.8 × 3.2 × 4.6 cm rim enhancing centrally necrotic mass in the right breast (arrow). Biopsy of this lesion yielded invasive ductal carcinoma. The enhancing axillary lymph nodes (dashed arrow) are asymmetric compared to the contralateral side, and was biopsy-proven metastatic disease. The right breast is asymmetrically decreased in size. (**b**) Contrast-enhanced axial T1-weighted fat-suppressed image demonstrates the same rim-enhancing mass in the right breast (arrow). (**c**) Contrast-enhanced axial T1-weighted fat-suppressed image demonstrates an irregular 2.8 × 2.0 cm spiculated mass, compatible with metastatic lymph nodes (dashed arrow). (**d**) Contrast-enhanced axial T2-weighted fat-suppressed image demonstrates the same right breast mass (arrow) with associated skin thickening (*), compatible with skin involvement. Associated fibrosis (F) results in nipple retraction, indicating nipple involvement. (**e**) Non-contrast-enhanced T1-weighted axial image without fat suppression redemonstrates the mass (arrow), skin thickening (*) and fibrosis (F) resulting in nipple retraction. In addition to the above sequences, a full protocol MRI would also include a T1-weighted axial noncontrast image with fat suppression, at least three other dynamic axial T1-weighted post-contrast sequences, and a sagittal delayed post-contrast sequence.

Abbreviated breast MRI (abMRI) has been developed to increase access and decrease the cost of screening breast MRI. AbMRI consists of fewer sequences requiring a shorter time and lower cost for the examination. Multiple studies have investigated whether abMRI can replace a fpMRI in the screening setting. A prospective study by Kuhl et al. [69] demonstrated that abMRI was equal to fpMRI in sensitivity and specificity. In addition, their study demonstrated that abMRI significantly decreased MRI acquisition time and average time for radiologist interpretation without sacrificing the examination’s specificity or positive predictive value (abMRI 94.3% and 24.4%, respectively versus fpMRI 93.9% and 23.4%, respectively) [69]. The abMRI negative predictive value was 99.8% [69]. Osei et al. found comparable cancer detection and patient recall rates for abMRI vs. fpMRI, while abMRI significantly decreased the radiologist’s time for interpretation [70]. Weinstein et al. demonstrated screening abMRI resulted in a CDR of 27.4/1000 in women with dense breasts after a negative digital breast tomosynthesis [71]. This is in contrast to the reported CDR of an fpMRI in women with dense breasts of 16.5/1000 [30]. Although quite a number of studies have demonstrated the benefits of abMRI [72,73], a universal abMRI protocol has not yet been developed. It is hoped that once a protocol has been standardized, women with dense breasts and those at intermediate risk for breast cancer will have access to MRI as the most sensitive screening tool, rather than only patients at the highest risk [30,72,74]. An example abMRI protocol, as outlined in the American Journal of Roentgenology Expert Panel Review [74], is shown in Figure 5.

A major barrier to implementation of a population-wide abMRI screening program has been cost. The average insurance reimbursement for a fpMRI is $1084. Studies have demonstrated that 8% of screening MRIs detect a suspicious lesion (BIRADS 4 or 5) requiring additional workup and possible biopsy [75]. By way of contrast, abMRI, while not covered by insurance, can cost as low as $299. The lower cost is possible due to the decreased examination time, allowing for a greater volume and a shorter interpretation time. It would be optimal to further decrease the MRI time by facilitating quicker patient preparation. Current MRI machines are not designed for high patient throughput. Time preparing the scanner and maneuvering the patient into the magnet takes longer than abMRI image acquisition itself [76]. Screening-focused MRI magnets would allow for even greater throughput, more widespread availability, and lower cost for this lifesaving tool in the high-risk population. Ultimately, these new machines could be used for other types of abMRIs tailored to answer specific clinical questions [76].

Given that the cost of abMRI can be as low as $299, which is lower than the insurance copay for a fpMRI, some institutions have developed robust MRI high-risk screening programs using a self-pay model. Grimm et al. [74] recommend that institutions price their self-pay abMRIs as low as possible, so as to not create significant healthcare disparity.

In order to implement wider use of abMRI, a standardized abMRI protocol is needed. Future steps will likely include development of universal abMRI protocols as well as quality measures and guidelines. Subsequent studies will undoubtedly further define the role of abMRI in screening guidelines [76].

## 7. Conclusions

MRI is the most sensitive, currently available method for the detection of breast cancer. Its indications include screening high-risk populations, evaluating women with newly diagnosed breast cancer for extent of disease and planning surgical management, and assessing response to neoadjuvant chemotherapy. Currently developing indications include using abMRI to expand the availability of screening MRI to other high-risk populations. Breast MRI may also be used as a biomarker for breast cancer risk and efficacy of tamoxifen therapy as hormonal treatment for women with breast cancer, and as a risk reduction strategy for women at increased risk of breast cancer.

## Data Availability

Not applicable.
